# Investigating the Association Between Mean Arterial Pressure on 28-Day Mortality Risk in Patients With Sepsis: Retrospective Cohort Study Based on the MIMIC-IV Database

**DOI:** 10.2196/63291

**Published:** 2025-03-05

**Authors:** Qimin Chen, Wei Li, Ying Wang, Xianjun Chen, Dehua He, Ming Liu, Jia Yuan, Chuan Xiao, Qing Li, Lu Chen, Feng Shen

**Affiliations:** 1 Department of Critical Care Medicine, The Affiliated Hospital of Guizhou Medical University, Beijing Road No. 8, Yunyan District, Guiyang, Guizhou Province, 550001, China, 86 13511999117; 2 Clinical Trials Centre, The Affiliated Hospital of Guizhou Medical University, Guiyang, China

**Keywords:** mean arterial pressure, 28-day mortality, sepsis, MIMIC-Ⅳ, retrospective study, Medical Information Mart for Intensive Care IV

## Abstract

**Background:**

Sepsis is a globally recognized health issue that continues to contribute significantly to mortality and morbidity in intensive care units (ICUs). The association between mean arterial pressure (MAP) and prognosis among patients with patients is yet to be demonstrated.

**Objective:**

The aim of this study was to explore the association between MAP and 28-day mortality in ICU patients with sepsis using data from a large, multicenter database.

**Methods:**

This is a retrospective cohort study. We extracted data of 35,010 patients with sepsis from the MIMIC-IV (Medical Information Mart for Intensive Care) database between 2008 and 2019, according to the Sepsis 3.0 diagnostic criteria. The MAP was calculated as the average of the highest and lowest readings within the first 24 hours of ICU admission, and patients were divided into 4 groups based on the mean MAP, using the quadruple classification approach. Other worst-case indications from the first 24 hours of ICU admission, such as vital signs, severity of illness scores, laboratory indicators, and therapies, were also gathered as baseline data. The independent effects of MAP on 28-day mortality were explored using binary logistic regression and a two-piecewise linear model, with MAP as the exposure and 28-day mortality as the outcome variables, respectively. To address the nonlinearity relationship, curve fitting and a threshold effect analysis were performed.

**Results:**

A total of 34,981 patients with sepsis were included in the final analysis, the mean age was 66.67 (SD 16.01) years, and the 28-day mortality rate was 16.27% (5691/34,981). The generalized additive model and smoothed curve fitting found a U-shaped relationship between MAP and 28-day mortality in these patients. The recursive algorithm determined the low and high inflection points as 70 mm and 82 mm Hg, respectively. Our data demonstrated that MAP was negatively associated with 28-day mortality in the range of 34.05 mm Hg-69.34 mm Hg (odds ratio [OR] 0.93, 95% CI 0.92-0.94; *P*<.001); however, once the MAP exceeded 82 mm Hg, a positive association existed between MAP and 28-day mortality of patients with sepsis (OR 1.01; 95% CI 1.01-1.02, *P*=.002).

**Conclusions:**

There is a U-shaped association between MAP and the probability of 28-day mortality in patients with sepsis. Both the lower and higher MAP were related with a higher risk of mortality in patients with sepsis. These patients have a decreased risk of mortality when their MAP remains between 70 and 82 mm Hg.

## Introduction

Sepsis is a life-threatening organ dysfunction caused by a dysregulated host response to infection [1-3]. While mortality from sepsis has decreased over time after age standardization, it remains high [2-6]. According to the Global Burden of Disease Study, 48.9 million cases of sepsis were reported worldwide in 2017, accounting for 11 million deaths [7]. Additionally, sepsis is the most expensive disease to treat in the United States, costing $23.7 billion annually and accounting for 6.2 percent of all hospital admissions [8]. In low-income nations with a high sepsis burden, this cost could be significantly greater [9,10]. Sepsis has thus emerged as a serious public health concern with substantial global implications and economic cost.

Despite significant attempts to provide novel organ support strategies and identify the underlying etiology of sepsis, the mortality rate remains high [5,9]. Research indicates that early treatment of sepsis can improve prognosis [1,11]. Therefore, early detection of possible risk factors is crucial for patients with a poor prognosis [12]. Mean arterial pressure (MAP) is one of the most commonly used parameters for the evaluation of sepsis severity [13]. MAP is the driving pressure of tissue perfusion and plays a key role in maintaining the perfusion of tissue and organs. While autoregulation of regional perfusion may protect vital organs such as the brain or kidney from systemic hypotension, tissue perfusion becomes linearly dependent on arterial pressure below a certain MAP (approximately 60 mm Hg) [13]. However, the optimal range of MAP during sepsis resuscitation remains undetermined. The 2016 and 2021 SSC Surviving Sepsis Campaign guidelines recommend that MAP be maintained at ≥65 mm Hg in septic shock [14,15] for improving tissue perfusion and prognosis [14,15]. However, a study by Vincent et al [16] found that MAP did not have a linear relationship with ICU mortality, and several other studies have shown that increased MAP does not necessarily improve clinical outcomes in patients with sepsis or septic shock [13,17-22]. On the contrary, excessively high blood pressure may increase the incidence of adverse events [17].

Given that the appropriate blood pressure range is yet to be determined in patients with sepsis, and considering limitations in sample sizes, differences in study design, variability in covariate adjustments, and the heterogeneity of patient populations included in previous studies, it is necessary to study and identify the appropriate blood pressure range for patients with sepsis based on large databases. Therefore, our study aims to investigate the relationship between MAP and the 28-day risk of mortality in patients with sepsis using Medical Information Mart for Intensive Care (MIMIC-IV), a large sample of US sepsis databases, to identify the inflection point value associated with a lower 28-day risk of death in sepsis. This large sample size will provide more stable and reliable results, allowing us to gain a better understanding of the relationship between MAP and the 28-day risk of mortality in sepsis.

## Methods

### Study Design and Setting

This retrospective cohort study analyzed data from the MIMIC-IV database, including patients admitted to Beth Israel Deaconess Medical Center between 2008 and 2019.Our study complied with the RECORD (Research Reports Using Observational Routine Collection of Health Data) statement [23].

### Participants

The study population comprised adult patients with sepsis identified through *ICD-9* (*99,591‐99,592*) and *ICD-10* (*R652*, *R6520*, *R6521*) diagnostic codes. Inclusion criteria were patients aged ≥18 years and with a confirmed sepsis diagnosis. Patients lacking MAP data were excluded. Of the initial 377,207 records, 34,981 patients met the eligibility criteria and were included in the final analysis.

### Data Collection and Variables

Data extraction was performed by certified researchers following standardized MIMIC-IV database procedures. The primary exposure variable, MAP, was calculated as the average of highest and lowest readings within 24 hours of ICU admission. While MAP values naturally fluctuate during the course of sepsis, we chose this approach to provide clinically actionable targets, acknowledging that a more complex time-varying analysis might capture additional nuances in the relationship between MAP and outcomes. MAP was analyzed both as a continuous variable and categorized into quartiles (Q1: 34.05‐69.34 mm Hg; Q2: 69.34‐74.94 mm Hg; Q3: 74.94‐81.87 mm Hg; and Q4: 81.87‐159.47 mm Hg). The primary outcome was 28-day all-cause mortality, recorded as a binary variable (1=death, 0=survival).

Covariates were selected based on clinical relevance and previous literature [3,24-26]: demographic characteristics (gender, age, and race)，vital signs (heart rate, respiratory rate, and temperature)，laboratory measurements (eg, lactate)，disease severity indices such as Charlson Comorbidity Index, sequential organ failure assessment (SOFA) score, and acute physiology and chronic health evaluation (APACHE III score)，therapeutic interventions (ie, mechanical ventilation, renal replacement therapy, corticosteroids, vasoactive drugs, immunoglobulin, and antibiotics).

### Statistical Analysis

The analytical approach comprised three sequential steps. First, we constructed univariate and multivariate binary logistic regression models with progressive covariate adjustment: model 1 (unadjusted), model 2 (adjusted for demographics), and model 3 (fully adjusted for all covariates). Continuous variables were presented as mean (SD) or median (range), and categorical variables as frequencies (percentages). Second, we employed generalized additive models (GAM) with smooth curve fitting to evaluate potential nonlinear relationships between MAP and mortality. For identified nonlinear associations, we determined inflection points using recursive algorithms and constructed piecewise linear regression models. Third, we conducted sensitivity analyses using alternative outcome measures (30-day mortality), different MAP calculation methods (median values), and varying covariate selection approaches. All analyses were performed using R software (version 4.3.2; R Foundation for Statistical Computing) and Empower Stats software (X&Y Solutions, Inc. Boston, MA) [27], with statistical significance set at *P*<.05 (two-sided).

### Missing Data Handling

Patients with missing values for any of the following key variables were excluded from the analysis: MAP measurements, age, SOFA score, lactate levels, and mortality outcomes. To assess potential selection bias, we compared the characteristics of included and excluded patients.

### Ethical Considerations

The study protocol received approval from the institutional review boards of Beth Israel Deaconess Medical Center (2001-P-001699/14) and Massachusetts Institute of Technology (0403000206). The data used in this study were obtained from the MIMIC-IV database, which is a freely accessible, deidentified public database [28]. Given the deidentified nature of the database, the requirement for informed consent was waived. Participants were not financially compensated, as the study used data from publicly accessible databases rather than data provided directly by participants.

## Results

### Study Population Screening

**Figure 1.**
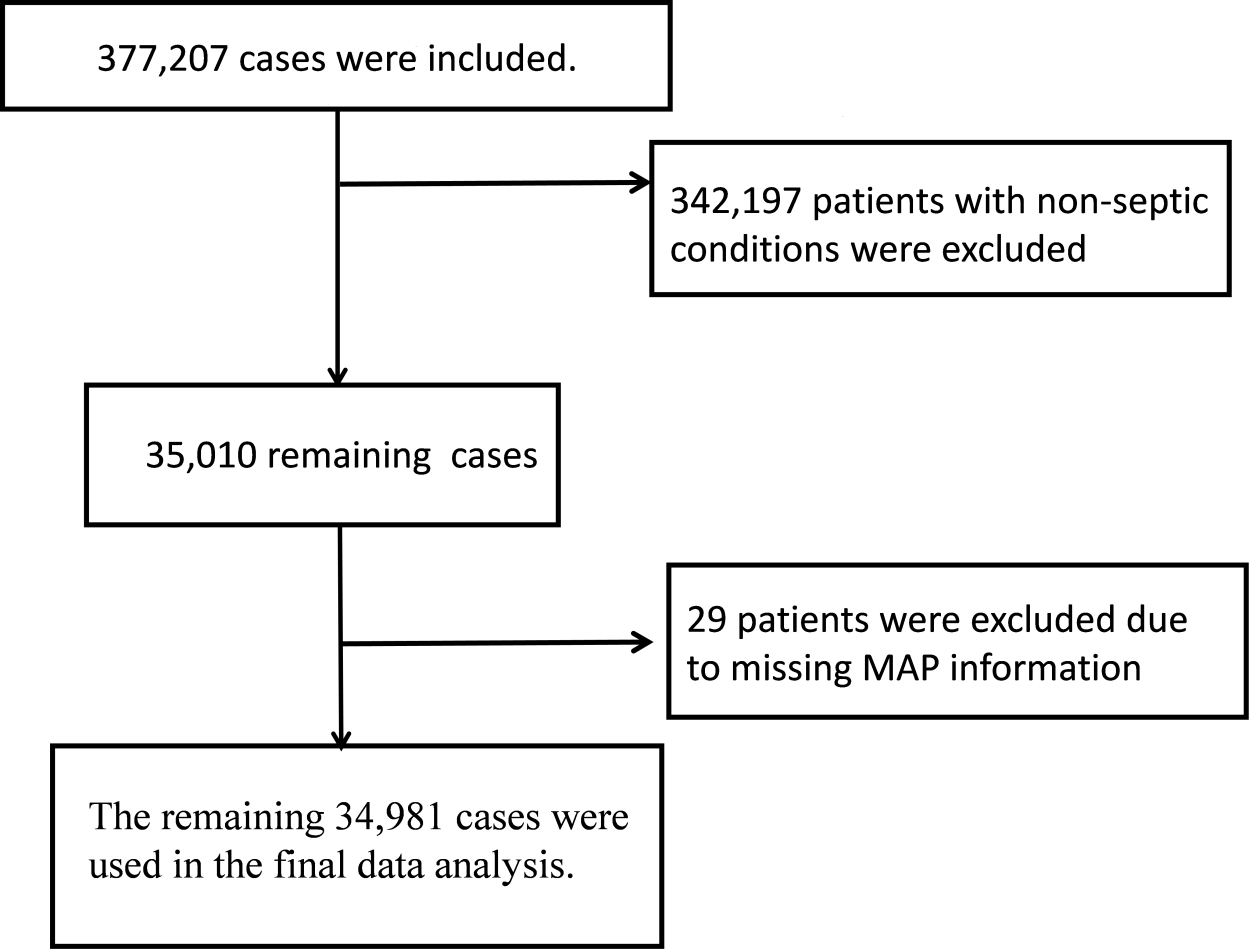
The flowchart of study. MAP: mean arterial pressure.

This study initially comprised 377,207 patients, of which 342,197 patients without sepsis and 29 patients lacking MAP data were excluded, with 34,981 cases remaining for final data analysis. The patient selection process is shown in the flowchart (Figure 1). Missing data were minimal (<5%, range 0‐4.1%) across all variables. Given the low proportion of missing data, a complete case analysis was employed. The validity of this approach was confirmed by comparison of baseline characteristics between included and excluded patients, revealing no significant differences in key features.

### Baseline Characteristics

Among the 34,981 included patients, 20,171 (57.66%) were men. The patients age was 66.67 (SD 16.01) years and the overall incidence of death within 28 days was 16.27% (5691/34,981). The patients’ baseline characteristics are summarized in Table 1. Patients were divided into 4 groups based on MAP at admission. The distribution of intravenous immunoglobulin use did not differ significantly among the MAP subgroups (*P*=.09). Compared with the other three groups (Q2-Q4), patients in the Q1 group were older, had higher age, Charlson Comorbidity Index, SOFA scores, APACHE III scores, and lactate levels. They also had a higher frequency of renal replacement therapy, and use of norepinephrine, dopamine, dobutamine, cortisone, and antibiotics such as carbapenem, cephalosporin, and vancomycin. These characteristics, which are associated with poor prognosis, explain why patients in the Q1 group had the highest 28-day mortality rate. In contrast, as MAP increased from Q1 to Q4, age of the patient, severity of the disease, and the proportion of patients using relevant medications progressively decreased between groups. It may be a potential mechanism for the observed differences in 28-day mortality risk across MAP levels.

**Table 1.** Baseline characteristics of patients with sepsis in the MIMIC-IV database, 2008‐2019.

Variables	Q1^a^ (n=8745)		Q2^a^ (n=8744)	Q3^a^ (n=8745)	Q4^a^ (n=8747)	*P* value
MAP^b^ range (mm Hg)	(34.05‐69.34)		(69.34‐74.94)	(74.95‐81.87)	(81.87‐159.47)	–^c^
Age (years), mean (SD)	71.72 (15.36)		69.76 (15.09)	66.92 (15.33)	64.67 (16.39)	<.001
Males, n (%)	4687 (53.60)		5178 (59.22)	5180 (59.23)	5126 (58.60)	<.001
White population, n (%)	6350 (72.61)		6166 (70.52)	5860 (67.01)	5268 (60.23)	<.001
Charlson comorbidity index, mean (SD)	6.86 (2.83)		6.11 (2.87)	5.87 (2.92)	5.62 (3)	<.001
SOFA^e^, mean (SD)	7.47 (4.02)		6.88 (3.79)	6.37 (3.56)	5.52 (3.22)	<.001
APACHE III^d^, mean (SD)	81.43 (27.98)		72.11 (27.49)	67.21 (26.55)	64.24 (24.95)	<.001
Lactate (mmol/L), mean (SD)	3.55 (3.46)		3.14 (2.78)	3.00 (2.75)	2.84 (2.56)	<.001
Heart rate (bpm), mean (SD)	100.76 (25.11)		103.01 (24.08)	104.63 (24.15)	107.46 (24.23)	<.001
Respiratory rate (bpm), mean (SD)	26.67 (9.60)		26.57 (9.67)	26.49 (9.62)	27.08 (9.71)	<.001
Temperature (°C), mean (SD)	36.68 (1.28)		36.73 (1.30)	36.77 (1.25)	36.92 (1.25)	<.001
Dexamethasone, n (%)						<.001
No	7907 (90.42)		7917 (90.54)	7841 (89.66)	7497 (85.71)	
Yes	838 (9.58)		827 (9.46)	904 (10.34)	1250 (14.29)	
Methylprednisolone, n (%)						<.001
No	7297 (83.44)		7322 (83.74)	7315 (83.65)	6909 (78.99)	
Yes	1448 (16.56)		1422 (16.26)	1430 (16.35)	1838 (21.01)	
Cortisone, n (%)						.002
No	8526 (97.50)		8557 (97.86)	8577 (98.08)	8596 (98.27)	
Yes	219 (2.50)		187 (2.14)	168 (1.92)	151 (1.73)	
Norepinephrine, n (%)						<.001
No	4868 (55.67)		5520 (63.13)	6274 (71.74)	7240 (82.77)	
Yes	3877 (44.33)		3224 (36.87)	2471 (28.26)	1507 (17.23)	
Dopamine, n (%)						<.001
No	7783 (89)		8155 (93.26)	8223 (94.03)	8364 (95.62)	
Yes	962 (11)		589 (6.74)	522 (5.97)	383 (4.38)	
Dobutamine, n (%)						<.001
No	8241 (94.24)		8363 (95.64)	8377 (95.79)	8504 (97.22)	
Yes	504 (5.76)		381 (4.36)	368 (4.21)	243 (2.78)	
IVIG^f^, n (%)						.09
No	8539 (97.64)		8538 (97.64)	8524 (97.47)	8495 (97.12)	
Yes	206 (2.36)		206 (2.36)	221 (2.53)	252 (2.88)	
MV^g^, n (%)						<.001
No	5335 (61.01)		4517 (51.66)	4502 (51.48)	5222 (59.70)	
Yes	3410 (38.99)		4227 (48.34)	4243 (48.52)	3525 (40.30)	
RRT^h^, n (%)						<.001
No	7879 (90.10）		8232 (94.14）	8281 (94.69）	8225 (94.03）	
Yes	866 (9.90）		512 (5.86）	464 (5.31）	522 (5.97）	
Carbapenem, n (%)						<.001
No	6458 (73.85)		6883 (78.72)	7053 (80.65)	7138 (81.61)	
Yes	2287 (26.15)		1861 (21.28)	1692 (19.35)	1609 (18.39)	
Cephalosporin, n (%)						<.001
No	7892 (90.25)		8008 (91.58)	8007 (91.56)	8046 (91.99)	
Yes	853 (9.75)		736 (8.42)	738 (8.44)	701 (8.01)	
Penicillin, n (%)						<.001
No	3864 (44.19)		4381 (50.10)	4462 (51.02)	4200 (48.02)	
Yes	4881 (55.81)		4363 (49.90)	4283 (48.98)	4547 (51.98)	
Vancomycin, n (%)						<.001
No	1127 (12.89)		1662 (19.01)	1861 (21.28)	1909 (21.82)	
Yes	7618 (87.11)		7082 (80.99)	6884 (78.72)	6838 (78.18)	
28-day mortality, n (%)						<.001
No	6687 (76.47)		7452 (85.22)	7564 (86.50)	7587 (86.74)	
Yes	2058 (23.53)		1292 (14.78)	1181 (13.50)	1160 (13.26)	

a Patients were divided into 4 groups (Q1, Q2, Q3, and Q4) based on the mean MAP, using the quadruple classification approach.

b MAP: mean arterial pressure.

c Not applicable

d APACHE III: Acute Physiology and Chronic Health Evaluation III.

e SOFA: Sequential Organ Failure Assessment.

f IVIG: intravenous immunoglobulin.

g MV: mechanical ventilation.

h RRT: renal replacement therapy.

### Relationship Between Mean Arterial Pressure and 28-Day Mortality

#### Univariate and Multivariate Analysis

We conducted univariate and multivariate analyses to investigate the relationship between MAP and 28-day mortality in patients with sepsis. Table 2 displays the results of the association between MAP and mortality in sepsis using various covariate adjustment strategies. In the nonadjusted model 1, each 1 mm Hg increase in MAP reduced the risk of 28-day mortality by 3% (odds ratio [OR] 0.97, 95% CI 0.971-0.974; *P<*.001). After adjusting for demographic factors such as gender, age, and White race, model 2 showed that the trend of OR was not altered (OR 0.97, 95% CI 0.97-0.98; *P<*.001). However, after adjusting for all covariables presented in Table 1, model 3 demonstrated that each 1 mm Hg increase in MAP resulted in a 1% decrease in the risk of 28 day-mortality (OR 0.99, 95% CI 0.99-1.00; *P<*.001). In model 3, the association between MAP and the probability of death in 28 days reduced to 1%, which was lower than the 3% decline seen in Models 1 and 2. This distinction was further explored and analyzed; we screened for covariates and discovered that SOFA score, age, and the Charlson comorbidity index had the greatest influence on the outcomes. The methodological rationale for this analysis was to include or exclude the effect of covariates on the regression coefficients of the main independent variables when constructing regression models. For example, in the unadjusted model, we included the main independent variable, MAP and found that the regression coefficient with the outcome was −0.0304. However, in the fully adjusted model, we added a number of covariates such as age, Charlson Comorbidity Index, SOFA score, and heart rate, the regression coefficient of MAP decreased to −0.0072 when these covariates were included (Multimedia Appendix 1). This implies that these factors “explain” or “mediate” the relationship between MAP and outcome to some extent. Specifically, age, Charlson index, and SOFA scores, which might reflect the patients’ baseline status and illness severity, contributed to the influence of MAP on outcome. This methodological aspect, namely the change in regression coefficients of the independent variables observed between the basic and full models, allows us to gain a deeper understanding of the complex link between independent factors and outcomes. It can help us uncover potential interactions or mediating effects that give a foundation for understanding study findings. In addition, we performed a sensitivity analysis to verify the robustness of our findings by converting the MAP from a continuous variable into categorical variables (quartiles) and performing a trend test. The Q1 MAP was used as a reference; therefore, the same association was observed (*P<*.001) (Table 2).

**Table 2.** Relationship between mean arterial pressure and 28-day mortality.

Exposure	Model 1^a^ (OR, 95% CI)	Model 2^b^ (OR, 95% CI)	Model 3^c^ (OR, 95% CI)	*P* value
MAP^d^ (mm Hg)	0.97 (0.97-0.97)	0.97 (0.97-0.98)	0.99 (0.99-1.00)	<.001
MAP^e^ quartiles (mm Hg)				
<65	1	1	1	
65‐70	0.60 (0.55,-0.66)	0.61 (0.56 -0.67)	0.65 (0.59- 0.73)	<.001
70‐80	0.41 (0.38-0.45)	0.42 (0.39- 0.46)	0.51 (0.46- 0.56)	<.001
80‐85	0.38 (0.34-0.43)	0.40 (0.36- 0.45)	0.58 (0.51- 0.66)	<.001
≥85	0.38 (0.35- 0.42)	0.42 (0.38-0.47)	0.74 (0.65- 0.83)	<.001
*P* value for trend	<.001	<.001	<.001	–^f^

a Model 1 unadjusted.

b Model 2 adjusted for gender, age at admission, White race.

c Model 3 adjusted for gender, age at admission, White race.

d MAP as a continuous variable.

e MAP as a categorical variable.

f Not applicable.

### Nonlinear Association Between MAP and 28-Day Mortality

**Figure 2.**
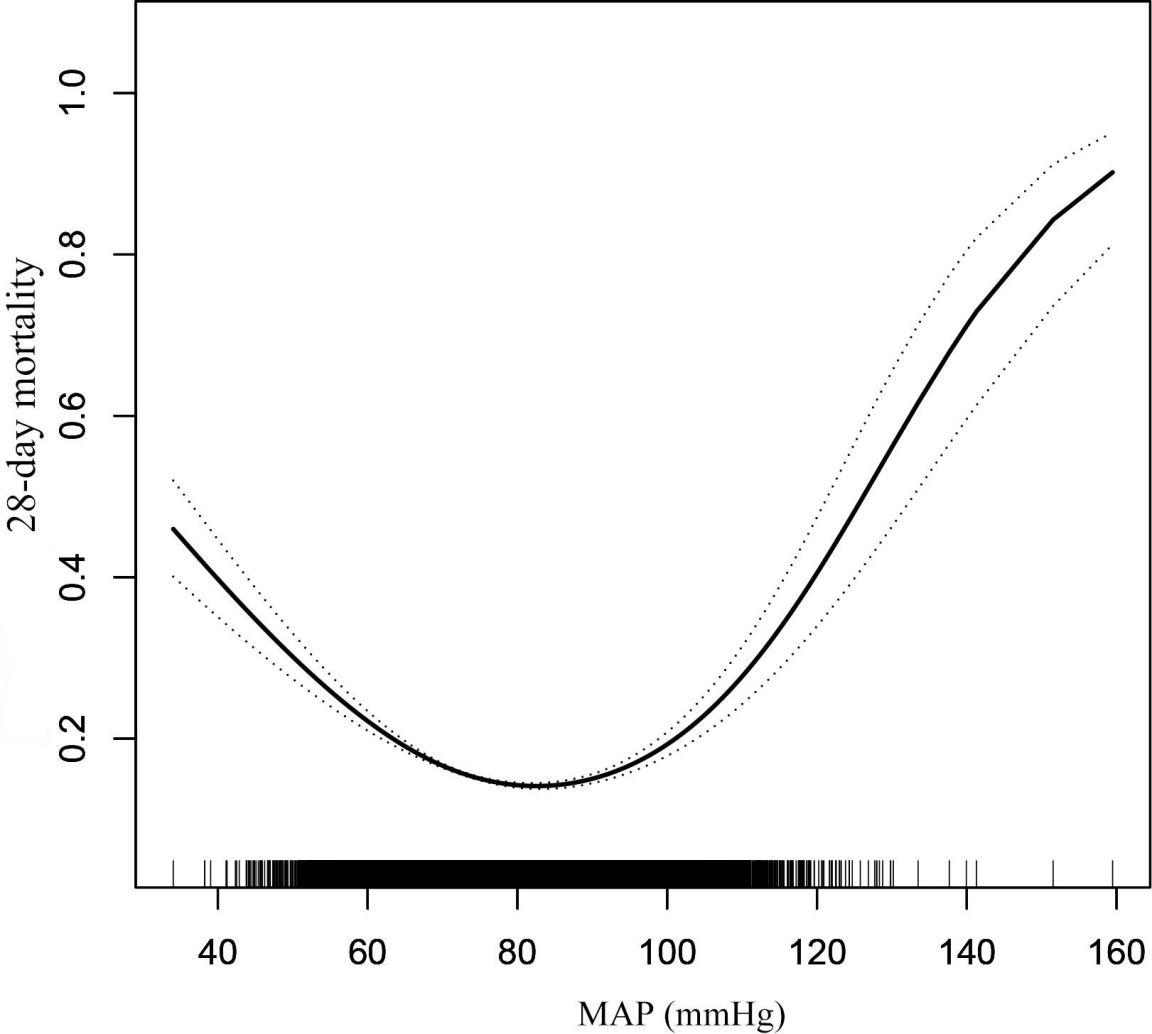
The nonlinear relationship between MAP and 28-day mortality risk. The solid line represents the smoothed curve fit while the dotted line represents 95% CI. MAP: mean arterial pressure.

We used smoothed curve fitting and GAM to evaluate the nonlinear relationship between MAP and 28-day mortality. After adjusting for all covariables listed in Table 1, our results demonstrated a U-shaped association between MAP and 28-day mortality with both low and high MAP associated with an increased risk of 28-day mortality (Figure 2). Furthermore, we employed a two-piecewise linear regression model and a recursive algorithm to determine the inflection points of MAP. The inflection points refer to points on the curve, where the curve transitions from falling to rising or rising to falling. Identifying these inflection points can help us better understand the complex relationship between MAP and 28-day mortality, as a single linear model may not fully capture this nonlinear relationship. We calculated the low and high inflection points at 70 and 82 mm Hg, respectively. When MAP was between 34.05 mm Hg and 70 mm Hg, the risk of 28-day mortality decreased by 7% for every 1 mm Hg increase (OR 0.93, 95% CI 0.92-0.94; *P*<.001). When the MAP was 70-82 mm Hg, there was no significant correlation (OR 1.01, 95% CI 0.99-1.02; *P*=.28). However, when MAP increased from 82 mm Hg to 159.47 mm Hg, the probability of 28-day mortality increased by 1% for every 1 mm Hg rise (OR 1.01, 95% CI 1.01-1.02, *P*=.002) (Table 3).

**Table 3.** Threshold effect analysis for the relationship between mean arterial pressure and 28-day mortality.

Outcomes	28-day mortality	
	OR (95%CI)	*P* value
Fitting model using logistic regression model	0.99 (0.99-1.00)	<.001
Fitting model using two-piecewise linear model		
Inflection point (mm Hg)		
<70	0.93 (0.92-0.94)	<.001
70‐82	1.01 (0.99-1.02)	.28
>82	1.01 (1.01-1.02)	.002
Log-likelihood ratio test		<.001

### Subgroup Analysis

The interaction analysis revealed significant effect modification by hypertension status (*P* for interaction=.03) and heart failure status (*P* for interaction=.03) on the association between MAP and 28-day mortality (Table 4). In hypertensive patients, each 1 mm Hg increase in MAP was associated with a slightly stronger protective effect against mortality (OR 0.993, 95% CI 0.992‐0.994) compared to nonhypertensive patients (OR 0.984, 95% CI 0.982‐0.986). Conversely, in patients with heart failure, the protective effect was marginally attenuated (OR 0.989, 95% CI 0.988‐0.990) compared to those without heart failure (OR 0.995, 95% CI 0.992‐0.997). The use of vasopressors did not significantly modify the association between MAP and mortality (*P* for interaction=.14), with similar protective effects observed in both vasopressor users (OR 0.988, 95% CI 0.984‐0.992) and nonusers (OR 0.991, 95% CI 0.990‐0.992). These findings suggest that the optimal blood pressure management strategy may need to be tailored according to patients’ comorbidity profile, particularly in those with hypertension or heart failure.

**Table 4.** Interaction analysis between mean blood pressure and treatment on 28-day mortality.

Model	28-day mortality, odds ratio (95% CI)	*P* value for interaction
Hypertension^a^		.03
No	0.984 (0.982-0.986)	
Yes	0.993 (0.992-0.994)	
Vasopressor^b^		.14
No	0.991 (0.990-0.992)	
Yes	0.988 (0.984-0.992)	
Heart failure^c^		.03
No	0.995 (0.992-0.997)	
Yes	0.989 (0.988-0.990)	

a Model Hypertension adjusted for: gender; age at admission; ethnicity; Charlson Comorbidity Index; dexamethasone; dopamine; dobutamine; IVIG, intravenous immunoglobulins; methylprednisolone; MV, mechanical ventilation; cortisone; SOFA, Sequential Organ Failure Assessment; glucocorticoids; cephalosporin; penicillin; heart rate; respiratory rate; vancomycin; body temperature and the interaction terms for following variables: dopamine, ethnicity, mechanical ventilation, SOFA.

b Model Vasopressor adjusted for: gender; age at admission; ethnicity; Charlson Comorbidity Index; dexamethasone; IVIG, intravenous immunoglobulins; methylprednisolone; MV, mechanical ventilation; cortisone; SOFA, Sequential Organ Failure Assessment; glucocorticoids; cephalosporin; penicillin; heart rate; respiratory rate; vancomycin; body temperature and the interaction terms for following variables: age at admission.

c Model Heart failure adjusted for: gender; age at admission; ethnicity; Charlson Comorbidity Index; dexamethasone; IVIG, intravenous immunoglobulins; methylprednisolone; MV, mechanical ventilation; cortisone; SOFA, Sequential Organ Failure Assessment; glucocorticoids; cephalosporin; penicillin; respiratory rate; vancomycin; body temperature and the interaction terms for following variables: age at admission, dexamethasone, IVIG, SOFA, glucocorticoids, cephalosporin, vancomycin, body temperature.

### Sensitivity Analysis

Our sensitivity trials yielded results that confirmed the robustness of our findings (Multimedia Appendix 2). The regression coefficient for MAP was –0.0068 (95% CI –0.0123 to –0.0013) when the endpoint was 30-day mortality. When the median was used, the regression coefficient for MAP was –0.0065 (95% CI –0.0118 to –0.0012). Following the exclusion of individuals receiving medication for hypertension, the MAP regression coefficient was –0.0070 (95% CI –0.0128 to –0.0012). Other covariate selection methods produced results that were comparable. These results support our main finding, which confirms that the chance of patient mortality is inversely correlated with the MAP in all conditions.

## Discussion

### Main Findings

We investigated the relationship between MAP and the 28-day risk of mortality in patients with sepsis using data from a large, multicenter retrospective cohort study in the United States. We found a nonlinear association between MAP and 28-day mortality using a GAM and a two-piecewise linear regression. The findings revealed a U-shaped connection, indicating that both high and low MAP was associated with an increased risk of 28-day mortality in patients with sepsis. We discovered that each 1 mm Hg increase in MAP below 70 mm Hg resulted in a 7% decrease in the 28-day risk of mortality. When MAP exceeded 82 mm Hg, the 28-day mortality risk increased by 1% for each 1 mm Hg rise. This implies that maintaining MAP between 70-82 mm Hg may help improve the outcome in individuals with sepsis. While previous studies, including the work by Zhong et al [29] have explored the relationship between MAP and sepsis outcomes using MIMIC-III data, our study extends these findings in several important ways. First, we used the updated MIMIC-IV database with a larger sample size (34,981 vs. 14,031), providing more statistical power. Second, we employed more sophisticated statistical methods, including GAM models and two-piecewise linear regression, which allowed us to identify specific inflection points. Most importantly, our study identified a precise optimal MAP range (70-82 mm Hg) for clinical practice, whereas previous studies were unable to determine such specific thresholds. These differences explain why our findings provide more detailed and clinically applicable guidance for MAP management in patients with sepsis. There is no substantial relationship between MAP and 28-day mortality in this range, and organ damage from hypoperfusion or hyper-perfusion can be avoided. This finding provides clinicians with a reference range that can help guide blood pressure management in patients with sepsis. Further, more clinical studies are needed to validate these findings. However, this study provides a valuable reference for optimizing blood pressure management in patients with sepsis.

Most previous studies have explored the effect of low MAP on the outcome in patients with sepsis; our study further discovered a U-shaped relationship with specific inflection points. Our findings regarding the lower MAP threshold (70 mm Hg) are consistent with several previous studies. In a single-center retrospective cohort study of 1395 patients with severe sepsis or septic shock, []the 28-day mortality rate with an average MAP<65 mm Hg was 39.7%, which was significantly higher than other groups [30]. Similarly, in another retrospective cohort study based on the MIMIC III database by Cao et al. [31], which included 14,607 patients with sepsis, it was found that a lower MAP was significantly associated with higher 30-day mortality rates, with the lowest inflection point at 68.6 mm Hg.

However, our study differs from previous research in several important aspects. First, we used more sophisticated statistical methods including GAM models and two-piecewise linear regression, enabling us to identify both lower and upper thresholds. Although previous studies comparing the effects of higher and lower MAP on sepsis outcomes have yielded negative results, our findings uniquely identified 82 mm Hg as a critical upper threshold, above which there is an increased risk of death [17,18]. This increased risk could be due to secondary damage caused by excessive workload on organs and ischemia-reperfusion injury [31,32].

We speculate that the disparities between our findings and previous studies can be attributed to several factors: (1) the populations studied were not identical; (2) we provided more comprehensive covariate adjustment, whereas previous studies did not adjust for potential confounders; and (3) our analytical approach using the GAM model allowed us to detect nonlinear associations that might have been overlooked in previous studies. Further research is needed to determine whether these findings can be applied across populations.

### Strengths and Limitations

This study has several key advantages. First, we used data from the updated MIMIC-IV cohort, which provides a larger sample size and, consequently greater statistical power. Second, we adjusted for a broader set of covariate indicators and focused on treatment strategies that were more closely related to patient outcomes. To better determine the true relationship between MAP and mortality, we used a GAM and two-piecewise linear models, which are advanced algorithms. Furthermore, we calculated the U-curve and identified two inflection points that provided a safe MAP range associated with lower mortality risk. Third, as this study used a large number of sensitivity analyses, the results are more robust. More informative, supporting evidence for MAP monitoring and clinical decision-making based on this metric is provided through this study.

Several limitations of this study should be noted. First, our findings are based solely on the MIMIC-IV database, which predominantly includes patients from a single geographic region and may not be representative of other populations. Given potential racial and ethnic differences in cardiovascular responses and outcomes, external validation of our findings in different 17 populations, particularly among Asian populations including Chinese patients, would be valuable. Such validation studies could help establish whether the optimal MAP range identified in our study is universally applicable or needs to be adjusted for different populations. Second, while we adjusted for measurable confounders, we could not account for unmeasurable confounding. Additionally, due to substantial missing data in lactate measurements, we were unable to conduct subgroup analyses based on lactate levels. This limitation prevents a deeper understanding of how the optimal MAP targets might differ among patients with varying degrees of tissue hypoperfusion, as indicated by lactate levels. Third, as this was an observational study, and therefore, inherently constrained, we could only observe relationships rather than evaluate causality.

### Conclusion

There is a U-shaped association between MAP and the mortality risk in patients with sepsis. Both increases or decreases in MAP are linked to increased mortality risk. Our findings suggest that patients with sepsis have a lower risk of death when their MAP is maintained between 70-82 mm Hg.

## Supplementary material

10.2196/63291 Multimedia Appendix 1 Baseline characteristics of patients with sepsis in the MIMIC-Ⅳdatabase, 2008-2019.

10.2196/63291 Multimedia Appendix 2 Relationship between mean arterial pressure and 28-day mortality.

## Abbreviations

APACHE III: Acute Physiology and Chronic Health Evaluation III
ICU: intensive care units
MAP: mean arterial pressure
MIMIC-IV: Medical Information Mart for Intensive Care
SOFA: sequential Organ Failure Assessment

## References

[1] Singer M, Deutschman CS, Seymour CW (2016). The third international consensus definitions for sepsis and septic shock (Sepsis-3). JAMA.

[2] Shankar-Hari M, Phillips GS, Levy ML (2016). Developing a new definition and assessing new clinical criteria for septic shock: for the third international consensus definitions for sepsis and septic shock (Sepsis-3). JAMA.

[3] Seymour CW, Liu VX, Iwashyna TJ (2016). Assessment of clinical criteria for sepsis: for the third international consensus definitions for sepsis and septic shock (Sepsis-3). JAMA.

[4] Vincent JL (2020). Highlighting the huge global burden of sepsis. Anaesth Crit Care Pain Med.

[5] Rhee C, Dantes R, Epstein L (2017). Incidence and trends of sepsis in US hospitals using clinical vs claims data, 2009-2014. JAMA.

[6] Prest J, Sathananthan M, Jeganathan N (2021). Current trends in sepsis-related mortality in the United States. Crit Care Med.

[7] Rudd KE, Johnson SC, Agesa KM (2020). Global, regional, and national sepsis incidence and mortality, 1990–2017: analysis for the Global Burden of Disease Study. The Lancet.

[8] Torio CM, Moore BJ (2016). Healthcare Cost and Utilization Project (HCUP) Statistical Briefs.

[9] Chiu C, Legrand M (2021). Epidemiology of sepsis and septic shock. Curr Opin Anaesthesiol.

[10] Vincent JL, Marshall JC, Namendys-Silva SA (2014). Assessment of the worldwide burden of critical illness: the intensive care over nations (ICON) audit. Lancet Respir Med.

[11] Wu M, Islam MM, Poly TN, Lin MC (2024). Application of AI in sepsis: citation network analysis and evidence synthesis. Interact J Med Res.

[12] Liang M, Xu Y, Ren X, Huang D, Jin M, Qiu Z (2024). The U-shaped association between serum osmolality and 28-day mortality in patients with sepsis: a retrospective cohort study. Infection.

[13] LeDoux D, Astiz ME, Carpati CM, Rackow EC (2000). Effects of perfusion pressure on tissue perfusion in septic shock. Crit Care Med.

[14] Rhodes A, Evans LE, Alhazzani W (2017). Surviving sepsis campaign: international guidelines for management of sepsis and septic shock: 2016. Intensive Care Med.

[15] Evans L, Rhodes A, Alhazzani W (2021). Surviving sepsis campaign: international guidelines for management of sepsis and septic shock 2021. Intensive Care Med.

[16] Vincent JL, Nielsen ND, Shapiro NI (2018). Mean arterial pressure and mortality in patients with distributive shock: a retrospective analysis of the MIMIC-III database. Ann Intensive Care.

[17] Asfar P, Meziani F, Hamel JF (2014). High versus low blood-pressure target in patients with septic shock. N Engl J Med.

[18] Lamontagne F, Richards-Belle A, Thomas K (2020). Effect of reduced exposure to vasopressors on 90-day mortality in older critically ill patients with vasodilatory hypotension: a randomized clinical trial. JAMA.

[19] Lamontagne F, Meade MO, Hébert PC (2016). Higher versus lower blood pressure targets for vasopressor therapy in shock: a multicentre pilot randomized controlled trial. Intensive Care Med.

[20] Thooft A, Favory R, Salgado DR (2011). Effects of changes in arterial pressure on organ perfusion during septic shock. Crit Care Lond Engl.

[21] Hylands M, Moller MH, Asfar P (2017). A systematic review of vasopressor blood pressure targets in critically ill adults with hypotension. Can J Anesth/J Can Anesth.

[22] Bourgoin A, Leone M, Delmas A, Garnier F, Albanèse J, Martin C (2005). Increasing mean arterial pressure in patients with septic shock: effects on oxygen variables and renal function. Crit Care Med.

[23] Benchimol EI, Smeeth L, Guttmann A (2015). The REporting of studies Conducted using Observational Routinely-collected health Data (RECORD) statement. PLoS Med.

[24] Roggeveen LF, Guo T, Fleuren LM (2022). Right dose, right now: bedside, real-time, data-driven, and personalised antibiotic dosing in critically ill patients with sepsis or septic shock-a two-centre randomised clinical trial. Crit Care.

[25] Maheshwari K, Nathanson BH, Munson SH (2018). The relationship between ICU hypotension and in-hospital mortality and morbidity in septic patients. Intensive Care Med.

[26] Burstein B, Tabi M, Barsness GW, Bell MR, Kashani K, Jentzer JC (2020). Association between mean arterial pressure during the first 24 hours and hospital mortality in patients with cardiogenic shock. Crit Care.

[27] EmpowerStats: simple & powerful tool for epidemiology and bio-statistics that helps you analyze data and create publishable result tables, all without writing code. EmpowerStats.

[28] Johnson AEW, Bulgarelli L, Shen L (2023). MIMIC-IV, a freely accessible electronic health record dataset. Sci Data.

[29] Zhong X, Li H, Chen Q (2023). Association between different MAP levels and 30-day mortality in sepsis patients: a propensity-score-matched, retrospective cohort study. BMC Anesthesiol.

[30] Lee GT, Hwang SY, Jo IJ (2019). Associations between mean arterial pressure and 28-day mortality according to the presence of hypertension or previous blood pressure level in critically ill sepsis patients. J Thorac Dis.

[31] Cao B, Chen Q, Tang T (2022). Non-linear relationship between baseline mean arterial pressure and 30-day mortality in patients with sepsis: a retrospective cohort study based on the MIMIC-III database. Ann Transl Med.

[32] Cai J, Chen X, Liu X (2022). AMPK: The key to ischemia-reperfusion injury. J Cell Physiol.

[33] Medical information mart for intensive care: freely available medical data for research. MIMIC.

