# Supplementary material for: Investigating the Association Between Mean Arterial Pressure on 28-Day Mortality Risk in Patients With Sepsis: Retrospective Cohort Study Based on the MIMIC-IV Database
Source: Interact J Med Res. 2025 Mar 5;14:e63291. doi: 10.2196/63291 (PMC11931324; doi:10.2196/63291)
Supplement: Multimedia Appendix 1 [file ijmr-v14-e63291-s001.docx]

|  | **Mean arterial pressure (mmHg)** | | | | |
| --- | --- | --- | --- | --- | --- |
| **Variables** | Q1 | Q2 | Q3 | Q4 | **P-value** |
|  | (34.05-69.34) | (69.34-74.94) | (74.95-81.87) | (81.87-159.47) |  |
| Patients, n | 8745 | 8744 | 8745 | 8747 |  |
| Age, (year) | 71.72 ± 15.36 | 69.76 ± 15.09 | 66.92 ± 15.33 | 64.67 ± 16.39 | <0.001 |
| Male，n(%) | 4687 (53.60) | 5178 (59.22) | 5180 (59.23) | 5126 (58.60) | <0.001 |
| White, n (%) | 6350 (72.61) | 6166 (70.52) | 5860 (67.01) | 5268 (60.23) | <0.001 |
| Charlson | 6.86 ± 2.83 | 6.11 ± 2.87 | 5.87 ± 2.92 | 5.62 ± 3.00 | <0.001 |
| SOFA | 7.47 ± 4.02 | 6.88 ± 3.79 | 6.37 ± 3.56 | 5.52 ± 3.22 | <0.001 |
| APACHE III | 81.43 ± 27.98 | 72.11 ± 27.49 | 67.21 ± 26.55 | 64.24 ± 24.95 | <0.001 |
| Lactate（mmol/L） | 3.55±3.46 | 3.14±2.78 | 3.00±2.75 | 2.84±2.56 | <0.001 |
| Heart rate (bpm) | 100.76 ± 25.11 | 103.01 ± 24.08 | 104.63 ± 24.15 | 107.46 ± 24.23 | <0.001 |
| Respiratory rate (bpm) | 26.67 ± 9.60 | 26.57 ± 9.67 | 26.49 ± 9.62 | 27.08 ± 9.71 | <0.001 |
| Temperature (°C) | 36.68 ± 1.28 | 36.73 ± 1.30 | 36.77 ± 1.25 | 36.92 ± 1.25 | <0.001 |
| Dexamethasone, n (%) |  |  |  |  | <0.001 |
| No | 7907 (90.42) | 7917 (90.54) | 7841 (89.66) | 7497 (85.71) |  |
| Yes | 838 (9.58) | 827 (9.46) | 904 (10.34) | 1250 (14.29) |  |
| Methylprednisolone, n (%) |  |  |  |  | <0.001 |
| Yes | 1448 (16.56) | 1422 (16.26) | 1430 (16.35) | 1838 (21.01) |  |
| Cortisone, n (%) |  |  |  |  | 0.002 |
| No | 8526 (97.50) | 8557 (97.86) | 8577 (98.08) | 8596 (98.27) |  |
| Yes | 219 (2.50) | 187 (2.14) | 168 (1.92) | 151 (1.73) |  |
| Norepinephrine, n (%) |  |  |  |  | <0.001 |
| No | 4868 (55.67) | 5520 (63.13) | 6274 (71.74) | 7240 (82.77) |  |
| Yes | 3877 (44.33) | 3224 (36.87) | 2471 (28.26) | 1507 (17.23) |  |
| Dopamine, n (%) |  |  |  |  | <0.001 |
| No | 7783 (89.00) | 8155 (93.26) | 8223 (94.03) | 8364 (95.62) |  |
| Yes | 962 (11.00) | 589 (6.74) | 522 (5.97) | 383 (4.38) |  |
| Dobutamine, n (%) |  |  |  |  | <0.001 |
| No | 8241 (94.24) | 8363 (95.64) | 8377 (95.79) | 8504 (97.22) |  |
| Yes | 504 (5.76) | 381 (4.36) | 368 (4.21) | 243 (2.78) |  |
| IVIG, n (%) |  |  |  |  | 0.089 |
| No | 8539 (97.64) | 8538 (97.64) | 8524 (97.47) | 8495 (97.12) |  |
| Yes | 206 (2.36) | 206 (2.36) | 221 (2.53) | 252 (2.88) |  |
| MV, n (%) |  |  |  |  | <0.001 |
| No | 5335 (61.01) | 4517 (51.66) | 4502 (51.48) | 5222 (59.70) |  |
| Yes | 3410 (38.99) | 4227 (48.34) | 4243 (48.52) | 3525 (40.30) |  |
| RRT, n (%) |  |  |  |  | <0.001 |
| No | 7879（90.10） | 8232（94.14） | 8281（94.69） | 8225（94.03） |  |
| Yes | 866（9.90） | 512（5.86） | 464（5.31） | 522（5.97） |  |
| Carbapenem, n (%) |  |  |  |  | <0.001 |
| No | 6458 (73.85) | 6883 (78.72) | 7053 (80.65) | 7138 (81.61) |  |
| Yes | 2287 (26.15) | 1861 (21.28) | 1692 (19.35) | 1609 (18.39) |  |
| Cephalosporin, n (%) |  |  |  |  | <0.001 |
| No | 7892 (90.25) | 8008 (91.58) | 8007 (91.56) | 8046 (91.99) |  |
| Yes | 853 (9.75) | 736 (8.42) | 738 (8.44) | 701 (8.01) |  |
| Penicillin, n (%) |  |  |  |  | <0.001 |
| No | 3864 (44.19) | 4381 (50.10) | 4462 (51.02) | 4200 (48.02) |  |
| Yes | 4881 (55.81) | 4363 (49.90) | 4283 (48.98) | 4547 (51.98) |  |
| Vancomycin, n (%) |  |  |  |  | <0.001 |
| No | 1127 (12.89) | 1662 (19.01) | 1861 (21.28) | 1909 (21.82) |  |
| Yes | 7618 (87.11) | 7082 (80.99) | 6884 (78.72) | 6838 (78.18) |  |
| 28-day mortality, n (%) |  |  |  |  | <0.001 |
| No | 6687 (76.47) | 7452 (85.22) | 7564 (86.50) | 7587 (86.74) |  |
| Yes | 2058 (23.53) | 1292 (14.78) | 1181 (13.50) | 1160 (13.26) |  |

Variables are presented as mean ± SD, median (IQR) or N (%)

APACHE III: Acute Physiology and Chronic Health Evaluation III; SOFA: Sequential Organ Failure Assessment; Charlson: Charlson comorbidity index; IVIG, intravenous immunoglobulin; MV, mechanical ventilation; RRT: renal replacement therapy.
