# Supplementary material for: Investigating the Association Between Mean Arterial Pressure on 28-Day Mortality Risk in Patients With Sepsis: Retrospective Cohort Study Based on the MIMIC-IV Database
Source: Interact J Med Res. 2025 Mar 5;14:e63291. doi: 10.2196/63291 (PMC11931324; doi:10.2196/63291)
Supplement: Multimedia Appendix 2 [file ijmr-v14-e63291-s002.docx]

| Exposure | ModelⅠ | ModelⅡ | Model Ⅲ |
| --- | --- | --- | --- |
|  | OR（95%CI，*P*） | OR（95%CI，*P*） | OR（95%CI，*P*） |
| ^*^MAP mmHg | 0.97 (0.97, 0.97) <0.0001 | 0.97 (0.97, 0.98) <0.0001 | 0.99 (0.99, 1.00) <0.0001 |
| ^#^MAP QUARTILE |  |  |  |
| <65 | 1.0 | 1.0 | 1.0 |
| 65-70 | 0.60 (0.55, 0.66) <0.0001 | 0.61 (0.56, 0.67) <0.0001 | 0.65 (0.59, 0.73) <0.0001 |
| 70-80 | 0.41 (0.38, 0.45) <0.0001 | 0.42 (0.39, 0.46) <0.0001 | 0.51 (0.46, 0.56) <0.0001 |
| 80-85 | 0.38 (0.34, 0.43) <0.0001 | 0.40 (0.36, 0.45) <0.0001 | 0.58 (0.51, 0.66) <0.0001 |
| ≥85 | 0.38 (0.35, 0.42) <0.0001 | 0.42 (0.38, 0.47) <0.0001 | 0.74 (0.65, 0.83) <0.0001 |
| P for trend | <0.0001 | <0.0001 | <0.0001 |

Relationship between mean arterial pressure and 28-day mortality.

*: MAP as a continuous variable.

^#^: MAP as a categorical variable.

ModelⅠadjust for: None

ModelⅡadjust for: gender; age at admission; white race.

ModelⅢadjust for: gender; age at admission; white race; Charlson Comorbidity Index; dexamethasone; methylprednisolone; cortisone; Norepinephrine; dopamine; dobutamine; lactate; IVIG, intravenous immunoglobulins; MV, mechanical ventilation; RRT: renal replacement therapy; SOFA, Sequential Organ Failure Assessment; APACHE III, Acute Physiology and Chronic Health Evaluation III; carbene penicillin; cephalosporin; penicillin; vancomycin; heart rate; respiratory rate; body temperature.
